# Wild Boar (*Sus scrofa*) as Reservoir of Pathogenic and Intermediate *Leptospira*

**DOI:** 10.3390/ani16071025

**Published:** 2026-03-27

**Authors:** Alice Stagnoli, Robert Valerio House, Karen Dohmann, Tomke Friederike Prüser, Anne-Catrin Geuthner, Catrin Albrecht, Martin Pfeffer

**Affiliations:** 1 Department of Veterinary Medicine, State Office for Consumer Protection Saxony-Anhalt, 39576 Stendal, Germany; alice.stagnoli@sachsen-anhalt.de (A.S.);; 2 Centre of Veterinary Public Health, Institute of Animal Hygiene and Veterinary Public Health, University of Leipzig, 04103 Leipzig, Germany; pfeffer@vetmed.uni-leipzig.de; 3 Department of Food Safety, State Office for Consumer Protection Saxony-Anhalt, 06112 Halle (Saale), Germany; tomke.prueser@sachsen-anhalt.de (T.F.P.);

**Keywords:** leptospirosis, wildlife, *Leptospira fainei*, intermediate *Leptospira*, *Sus scrofa*, Germany, zoonosis

## Abstract

Leptospirosis is a globally widespread zoonosis, with wildlife species playing a key role in the maintenance and environmental dissemination of *Leptospira* spp. Wild boar (*Sus scrofa*) are increasingly recognized as potential reservoir; however, molecular data on renal carriage of *Leptospira* spp. in Europe and particularly in Germany, remain scarce. In this study, 1281 wild boar kidney samples collected in the federal state of Saxony-Anhalt were analyzed by real-time PCR to investigate the presence of pathogenic and intermediate *Leptospira* DNA. Pathogenic *Leptospira* DNA was detected in 3.1% (95% CI = 2.3–4.2) of the samples, whereas intermediate *Leptospira* DNA was identified in 0.6% (95% CI = 0.3–1.2). Pathogenic *Leptospira* showed a widespread distribution across the study area, with a significantly higher prevalence in the district of Salzlandkreis (*p* = 0.04, OR = 3.4, CI = 0.8–10.3). Evaluation of host-related and environmental factors revealed a significant association between pathogenic *Leptospira* DNA detection and both sub-adult age class (χ^2^ = 13.1, *df* = 2, *p* = 0.0014) and autumn season (χ^2^ = 12.3, *df* = 3, *p* = 0.0066), whereas no significant association was observed for sex. Neither of these factors showed a significant relation with intermediate *Leptospira* spp. Sequence analyses of *rrs* and *16S rRNA* genes predominantly identified as *L. interrogans* (62.5%), followed by *L. borgpetersenii* (20.0%) and *L. kirschneri* (5.0%). All intermediate *Leptospira* sequences were identified as *L. fainei*. Notably, this study provides the first molecular evidence of intermediate *L. fainei* DNA in wild boar kidneys in Germany. Overall, these findings suggest that wild boar may act as renal carriers of *Leptospira* spp. and support their potential relevance in the epidemiology of leptospirosis within wildlife populations in Saxony-Anhalt.

## Simple Summary

Leptospirosis is a disease affecting both animals and humans, caused by bacteria that can persist in water and soil contaminated by animal urine. Wild animals, such as wild boar, can carry these bacteria in their kidneys and, in most cases, release them into the environment without showing any signs of illness, thereby contributing to the spread of the disease. In Europe, and particularly in Germany, most previous studies on leptospirosis in wild boar were based on blood antibody tests indicating past exposure but not current bacteria presence in the examined animal. In this study, kidney samples from wild boar collected in Saxony-Anhalt, a federal state in Germany, were examined to detect the bacterial genetic material using polymerase chain reaction (PCR). The results showed that a small but relevant proportion of wild boar harbored leptospires DNA in their kidneys, with higher detection rates in certain regions, in sub-adult animals, and during autumn. In addition, this study provides the first direct evidence in Germany of so-called intermediate leptospires in wild boar kidneys by reporting the presence of a specific genotype, *Leptospira fainei* (*L. fainei*). These findings improve our understanding of how leptospires circulate in the environment and highlight the importance of monitoring wildlife to protect animal as well as human health, and ecosystems within a One Health framework.

## 1. Introduction

Leptospirosis, a zoonotic disease of global importance, has long been associated with rodents and other wild species, which act as asymptomatic reservoirs of pathogenic *Leptospira*. The genus *Leptospira* is currently classified into pathogenic, intermediate and saprophytic species based on genetic and phenotypic characteristics [1]. While pathogenic species are recognized as the main agents responsible for leptospirosis in humans and animals [2], intermediate species have increasingly been detected in both clinical and environmental samples and are considered to have a potential, although still not fully clarified, pathogenic role [3].

Over the past decades, numerous investigations conducted worldwide [4,5,6] and especially in Germany [7,8,9,10] have documented the presence of these bacteria in the renal tissues of wild rodents, highlighting their pivotal role in environmental contamination and transmission of infection to domestic animals and humans [11,12]. In parallel, many studies have examined a variety of wild carnivores, shedding light on their potential role as carriers [13,14,15,16,17]. Despite this growing body of research, the wild boar (*Sus scrofa*) remains an understudied species in the context of *Leptospira* carriage. This ungulate, whose populations have markedly expanded across the European continent over recent decades [18], frequently roams at the interface between wildlife habitats, agricultural areas and human settlements. These ecological characteristics result in frequent contact with livestock, companion animals and humans, increasing the likelihood of cross-species transmission. Consequently, the wild boar may represent a key host in the epidemiology of *Leptospira* spp., potentially contributing to both environmental contamination and infection spread [19]. Nevertheless, the extent to which wild boar harbor pathogenic or intermediate *Leptospira* in their kidneys remains insufficiently explored. In particular, no study to date has specifically investigated the presence of intermediate *Leptospira* in wild boar kidneys in Germany.

Recent serological data collected in the federal state of Saxony-Anhalt, Germany, during 2023–2024 revealed that 12.4% of examined wild boar sera carried antibodies against *Leptospira* spp. including the intermediate species *L. fainei* serovar Hurstbridge [20]. These results suggest that exposure to intermediate leptospires does occur in the wild boar population of Saxony-Anhalt. However, serology alone cannot distinguish between past exposure and active renal infection. To date, evidence on the presence of pathogenic or intermediate *Leptospira* in the renal tissue of wild boar from Saxony-Anhalt is not available.

In light of this knowledge gap, targeted investigations are essential to clarify the actual role of wild boar in maintaining and potentially disseminating these pathogens across the landscape. Understanding whether these animals harbor bacteria in their kidneys is critical for assessing the risk they may pose to other wildlife species, domestic animals, hunters and people living or working in rural environments.

The present study aims to fill this knowledge gap by investigating the occurrence of pathogenic and intermediate *Leptospira* spp. in wild boar kidneys collected in Saxony-Anhalt, providing new insights into the epidemiological significance of this widespread ungulate species.

## 2. Materials and Methods

### 2.1. Sample Collection

Kidney samples of wild boar were collected from animals hunted during 2024. These animals were sampled as part of the routine monitoring program for Classical and African Swine Fever [21]. All kidney samples were obtained *post-mortem* by trained hunters using sterile instruments and thus minimizing all possible contamination. Hunters were supplied by our institute with standardized sampling kits, which included sterile sample bags, collection instructions and materials to maintain sample integrity during transport. For each animal, hunters recorded relevant data on a standardized form, including date of hunting, county, geographical coordinates of the hunting site, sex and age. The age was categorized into three classes: young (under 12 months), sub-adult (between 12 and 24 months) and adult (over 24 months), estimated based on dental eruption patterns [22], a method taught in mandatory hunter training courses.

Since sampling was conducted during the hunting season, the sample size could not be predicted beforehand.

**Figure 1:**
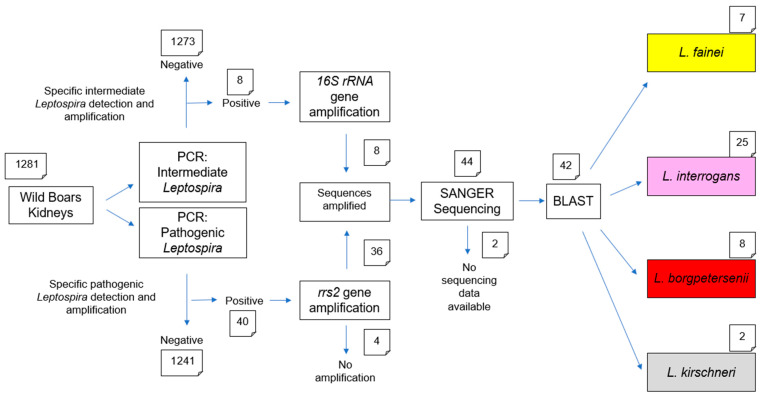
Workflow of the laboratory analyses of *L*. species including the sequential decision-making process used to obtain the final diagnostic outcomes.

Upon arrival at the laboratory, kidney samples were immediately processed. Samples showing signs of autolysis or insufficient tissue quantity were excluded from further analyses to ensure the quality of downstream molecular assays. A scheme of all examinations performed is given in Figure 1.

### 2.2. Sample Preparation

A total of 1304 kidney samples were submitted during 2024. Of these, 23 were excluded due to insufficient quantity or poor quality, resulting in 1281 samples included in the analyses. From each sample, approximately 1 g of kidney tissue was aseptically collected in total from three randomly selected sites of the renal cortex using sterile instruments. As *Leptospira* are known to colonize the renal tubules, this tissue is particularly relevant for their detection [23]. For animals from which both kidneys, or fragments of both kidneys, were available, tissue samples of both organs were pooled prior to processing.

All procedures were performed under a laminar-flow biosafety cabinet to maintain sterility. Cross-contamination between animals was avoided through the sequential preparation of individual kidneys and stringent disinfection measures after each preparation. The collected tissues were homogenized in 7 mL phosphate buffered salt solution (PAN Biotech GmbH; Aidenbach, Germany) in PrioGENIZER™ Homogenization Device (Thermo Fisher Scientific Inc., Waltham, MA, USA) and immediately subjected to DNA extraction.

### 2.3. DNA Extraction and Real-Time PCR

DNA was extracted using the IndiSpin Pathogen Kit (Indical Bioscience GmbH, Leipzig, Germany), according to the manufacturer’s instructions. Successful extraction and DNA integrity were verified by real-time PCR amplification of the beta-actin gene, confirming the presence of amplifiable host DNA and the absence of PCR inhibitors. Extracted DNA was divided into two aliquots: one was used right after preparation for PCR, while the other was stored at −20 °C as a reserve to ensure long-term integrity.

Detection of *Leptospira* DNA was carried out using two independent real-time PCR assays each targeting a different group of *Leptospira* spp. The first assay targeted the lipL32 gene, which is specific for DNA of pathogenic *Leptospira* spp. [24], while the second assay targeted the *16S rRNA* gene for the detection of intermediate *Leptospira* spp. [25]. Both assays were performed on all samples.

Positive controls consisted of DNA extracted from pure cultures of *L*. *interrogans* serogroup Copenhageni strain M20 for the lipL32 assay and *L*. *fainei* serogroup Hurstbridge strain BUT6 for the *16S rRNA* assay. Both strains were obtained from the World Organization for Animal Health (WOAH) Reference Laboratory for Leptospirosis Amsterdam University Medical Center, Department of Medical Microbiology and Infection Prevention Amsterdam, The Netherlands. Sterile DNase- and RNase-free distilled water was used as a negative control.

Each real-time PCR was prepared in a final volume of 25 μL. The lipL32 reaction mix consisted of 12.5 µL of 2× QuantiTect Probe PCR Master Mix (Qiagen, Hilden, Germany), 2 μL of each primer (100 pmol/µL; Table 1), 3.5 µL of DNase- and RNase-free distilled water and 5 μL of template DNA. The *16S rRNA* assay was prepared using 12.5 µL of 2× TaqMan Genotyping Master Mix (Applied Biosystems, Foster City, CA, USA), 1 μL of each primer (25 pmol/µL; Table 1), 0.3 µL of each probe (25 pmol/µL) 4.9 µL of DNase- and RNase-free distilled water and 5 μL of template DNA.

**Figure 2:**
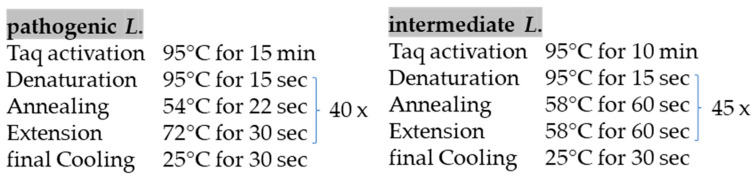
Summary of thermal cycling profiles for the real-time PCR assays used to detect pathogenic and intermediate *L.* spp., including activation, denaturation, annealing/extension temperatures, times and cycle numbers.

The real-time PCRs were run on AriaMx Real-Time PCR Systems (Agilent Technologies, Santa Clara, CA, USA) using the thermal cycling conditions adapted from previously published protocols [24,25], as detailed and shown in Figure 2.

### 2.4. Conventional PCR and Sanger Sequencing

Samples tested positive for DNA of pathogenic *Leptospira* by real-time PCR were further analyzed by conventional PCR (cPCR) targeting the *rrs2* gene, using primers described by Ahmed et al. [26], whereas samples positive for DNA of intermediate *Leptospira* were subjected to cPCR targeting the *16S rRNA* gene using primers modified in this study to better match with the DNA of intermediate *Leptospira* (Table 1). In addition, the positive controls used in the real-time PCR assays were included in the corresponding cPCR analyses in order to validate and confirm the applied methods.

**Figure 3:**
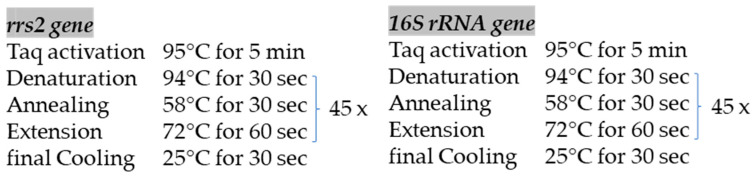
Summary of thermal cycling profiles for the cPCR assays used to detect DNA of pathogenic and intermediate *L.* spp., showing activation, denaturation, annealing/extension temperatures, times and cycle numbers.

The amplification of each target gene was performed using the HotStarTaq Master Mix Kit (Qiagen, Hilden, Germany) under thermal cycling conditions adapted from previously published protocols [26], as reported in Figure 3. PCR products were visualized by electrophoresis on a 1% agarose gel run at 100 V for 30 min and the size of the amplified DNA fragments was assessed under ultraviolet illumination.

Samples that were successfully amplified by cPCR were subsequently subjected to Sanger sequencing performed by SeqStudio Genetic Analyzer (Thermo Fisher Scientific, Waltham, MA, USA) using the same primers employed for the cPCR. Raw Sanger sequencing data were quality-checked, edited and aligned using MEGA11 software (Molecular Evolutionary Genetics Analysis; version 11.0.13). The resulting consensus sequences were compared with reference sequences available in the NCBI nucleotide database using the Basic Local Alignment Search Tool (version 2.17.0) for nucleotides (BLASTn) algorithm provided by the National Center of Biotechnology Information (NCBI) (Bethesda, MD, USA). The obtained sequences were deposited in GenBank under Acc. No. 15998750.

### 2.5. Statistical Analyses

The data were recorded in Microsoft Excel (Microsoft Office LTSC Standard 2021) and analyzed using Chi-square (χ^2^) and Fisher’s exact (F) tests using the R software packages (Version 4.5.1).

Statistical tests were applied to evaluate the association between *Leptospira* spp. infection status and season (winter, spring, summer and autumn), as well as between infection status and host-related factors, such as sex (male or female) and age (young, sub-adult and adult). Statistical significance threshold was set at *p* ≤ 0.05 [27].

Statistical analyses were performed separately for pathogenic and intermediate *Leptospira*. In the analysis of pathogenic *Leptospira*, samples tested positively for intermediate *Leptospira* were classified as negative, as they did not contain DNA belonging to the pathogenic group. Conversely, in the analysis of intermediate *Leptospira*, samples positive for pathogenic *Leptospira* were considered negative, as they were not positive for intermediate species.

## 3. Results

A total of 1281 wild boar kidney samples were analyzed by real-time PCR. Among these, DNA from pathogenic *Leptospira* was detected in 3.1% (40/1281) (95% CI = 2.3–4.2) of the samples, while 0.6% (8/1281) (95% CI = 0.32–1.23) tested positive for DNA of intermediate *Leptospira*. A detailed breakdown of the results, including the number of animals sampled in each age class, Ct values, as well as their distribution across the different counties, is presented in the Supplementary Materials (Tables S1–S4).

**Figure 4:**
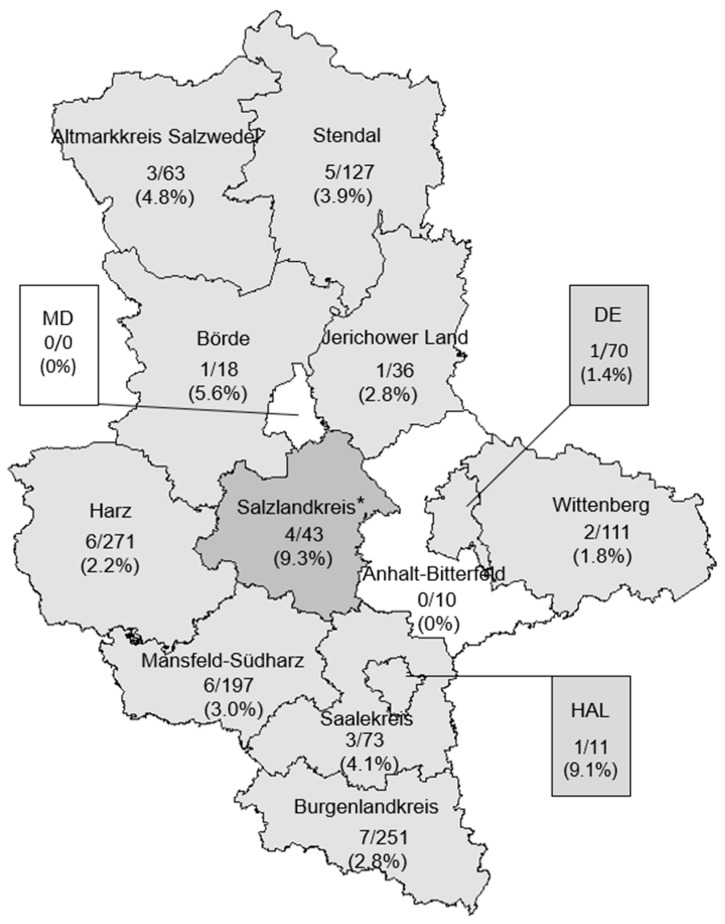
Prevalence of kidney samples tested positive for DNA of pathogenic *Leptospira* in wild boar from the different administrative districts of Saxony-Anhalt: DE = Dessau-Roßlau, MD = Magdeburg city, HAL = Halle (Saale). Statistically significant differences in the prevalence between individual districts and the overall prevalence in Saxony-Anhalt (3.1%) are indicated by an asterisk (*).

The spatial distribution of positive samples was evaluated at the administrative districts level. Samples positive for pathogenic *Leptospira* DNA were detected in all investigated administrative district of Saxony-Anhalt, with the exception of Anhalt-Bitterfeld and Magdeburg city. The highest number of positive samples was detected in the district of Salzlandkreis (9.3%) which was statistically significant when compared to the overall prevalence in Saxony-Anhalt (*p* = 0.04, OR = 3.4; CI = 0.8–10.3) (Figure 4).

**Figure 5:**
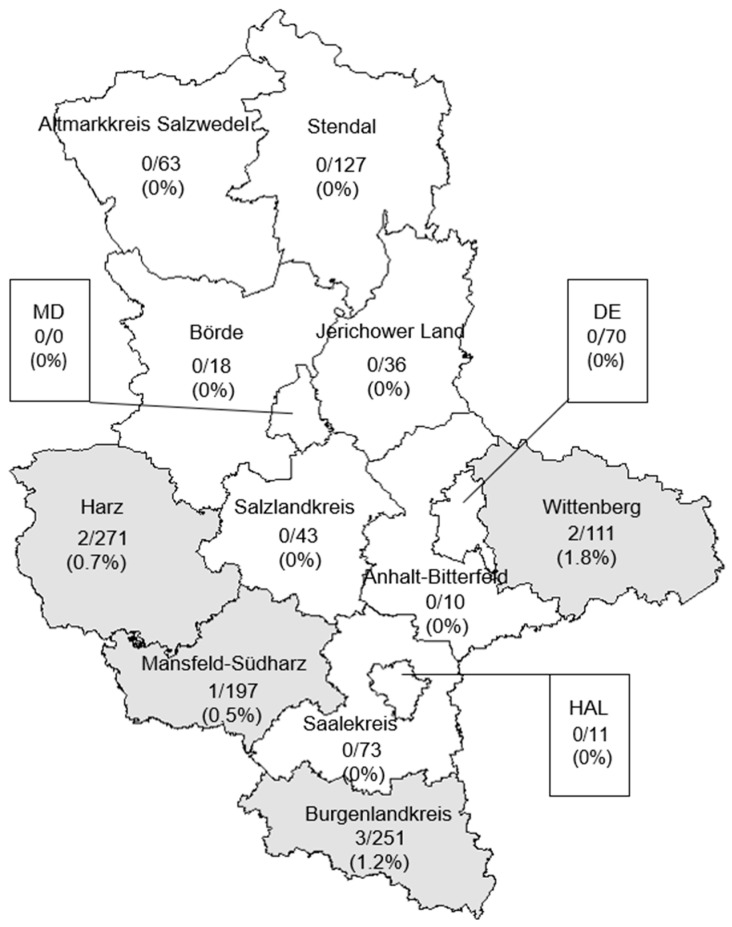
Prevalence of kidney samples tested positive for DNA of intermediate *Leptospira* in wild boar from the different administrative districts of Saxony-Anhalt: DE = Dessau-Roßlau, MD = Magdeburg city, HAL = Halle (Saale).

Concerning the intermediate *Leptospira* DNA, positive samples were identified in the districts of Mansfeld-Südharz (0.5%), Harz (0.7%), Burgenlandkreis (1.2%) and Wittenberg (1.8%), last one showing the highest prevalence. Comparison of the prevalence at the district level with the overall prevalence did not reveal statistically significant differences (Figure 5).

The potential influence of host-related factors, i.e., age and sex, as well as external factors, i.e., season, on the prevalence of pathogenic and intermediate *Leptospira* DNA in wild boar kidney samples was assessed.

Among the 1281 analyzed kidney samples, 745 originated from male wild boar, 490 from females and 46 from animals for which sex was not recorded by the hunters. Of the animals with unknown sex, 1 (2.2%) has been positively tested for pathogenic *Leptospira* DNA. Among animals with recorded sex, pathogenic *Leptospira* DNA was detected in 28 males (3.8%) and 11 females (2.2%), while intermediate *Leptospira* DNA was detected in 6 males (0.8%) and 2 females (0.4%). Overall, no statistically significant differences have been observed between males and females neither for pathogenic nor for intermediate *Leptospira*.

**Figure 6:**
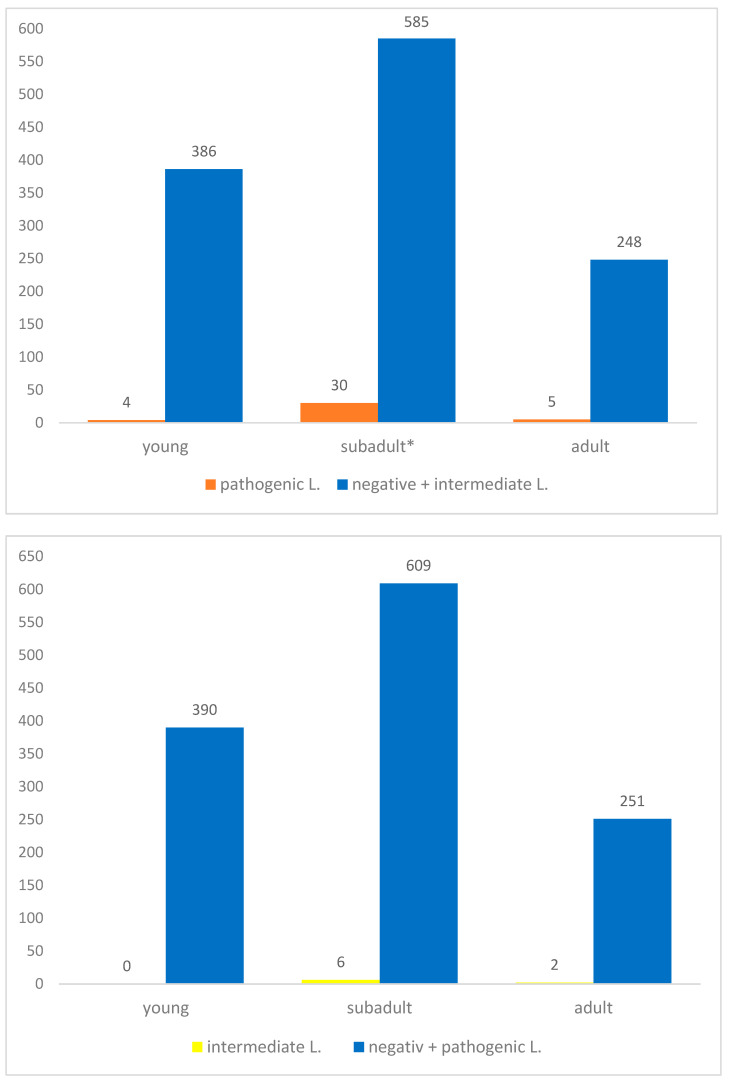
Total number of wild boar from Saxony-Anhalt stratified by age class and by negative and positive status for pathogenic (**upper panel**) and intermediate (**lower panel**) *L*. DNA. A statistically significant increase in the number of animals positive for pathogenic *L*. DNA was observed in the group of sub-adults and is indicated by an asterisk (*).

Considering the age of the examined animals, 390 (30.4%) were less than 1 year old, 615 (48.0%) were classified as sub-adults and 253 (19.8%) were adults. Age was not recorded for 23 (1.8%) wild boar, one of which (4.3%) tested PCR-positive for pathogenic *Leptospira* DNA. Pathogenic *Leptospira* DNA was detected in all three age groups, with the highest prevalence observed in sub-adult animals (30 PCR positive samples; 4.9%). This difference was statistically significant (χ^2^ = 13.1, *df* = 2, *p* = 0.0014) (Figure 6).

For intermediate *Leptospira*, no PCR-positive animals were detected among young animals, while positive samples were identified in sub-adults and adults, with the highest number of positive reactions observed in sub-adults (6; 1%). It was interesting to observe that intermediate *Leptospira* were only detected in the southern districts of the state; however, no statistically significant association was found between intermediate *Leptospira* positivity and district (Figure 5 and Figure 6).

**Figure 7:**
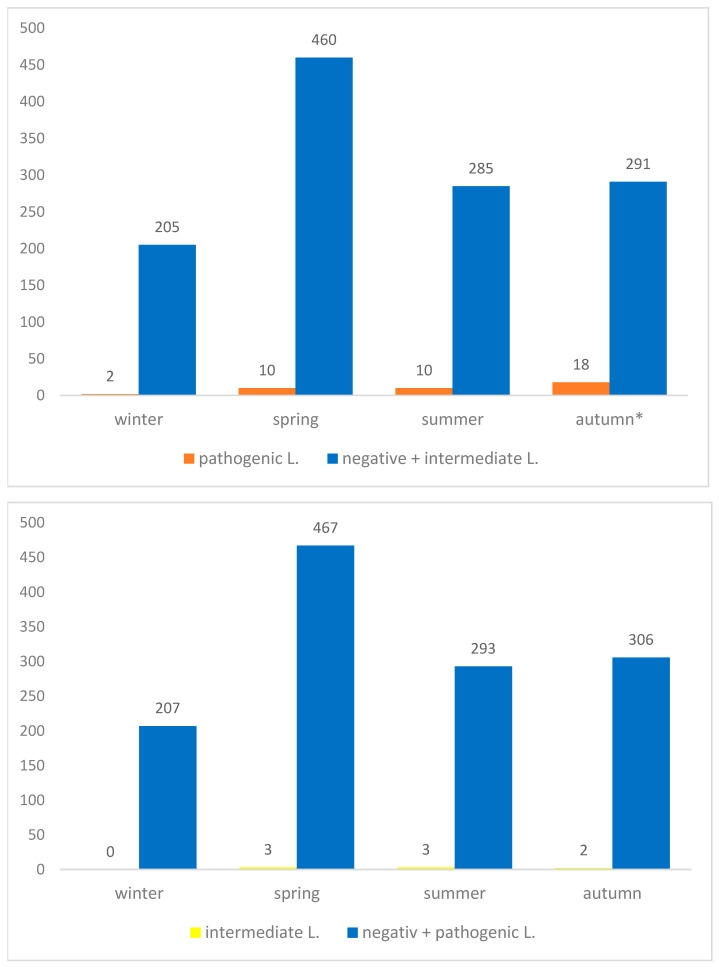
Total number of wild boar from Saxony-Anhalt stratified by season and by negative and positive status for pathogenic (**upper panel**) and intermediate (**lower panel**) *L*. DNA. A statistically significant increase was observed in autumn for pathogenic *Leptospira* indicated by an asterisk (*).

Considering the season of sample collection, 207 (16.2%) kidney samples were collected in winter, 470 (39.7%) in spring, 295 (23.0%) in summer and 309 (24.1%) in autumn. Regarding pathogenic *Leptospira*, PCR-positive samples were detected in all seasons, with 2 positive samples in winter (1.0%), 10 in spring (2.1%), 10 in summer (3.4%) and 18 in autumn (5.8%). The highest prevalence was observed in autumn, which differed significantly from the other seasons (χ^2^ = 12.3, *df* = 3, *p* = 0.0066) (Figure 7).

With regard to intermediate *Leptospira*, no PCR-positive samples were detected during winter, while positive samples were identified in spring (3; 0.6%), summer (3; 1%) and autumn (2; 0.6%). However, no statistically significant differences among seasons were observed for intermediate *Leptospira* DNA, as represented in Figure 7.

All 48 samples (40 positive for pathogenic *Leptospira* DNA and 8 positive for intermediate *Leptospira* DNA) identified by real-time PCR were subsequently subjected to cPCR using respective primer sets (Figure 3). Amplification products suitable for sequencing were obtained from 44 samples, including 36 pathogenic and all 8 intermediate *Leptospira* samples. Two samples (one pathogenic and one intermediate) were excluded from BLASTn analysis due to insufficient sequence quality, resulting in a total of 42 sequences included in the final analysis.

**Figure 8:**
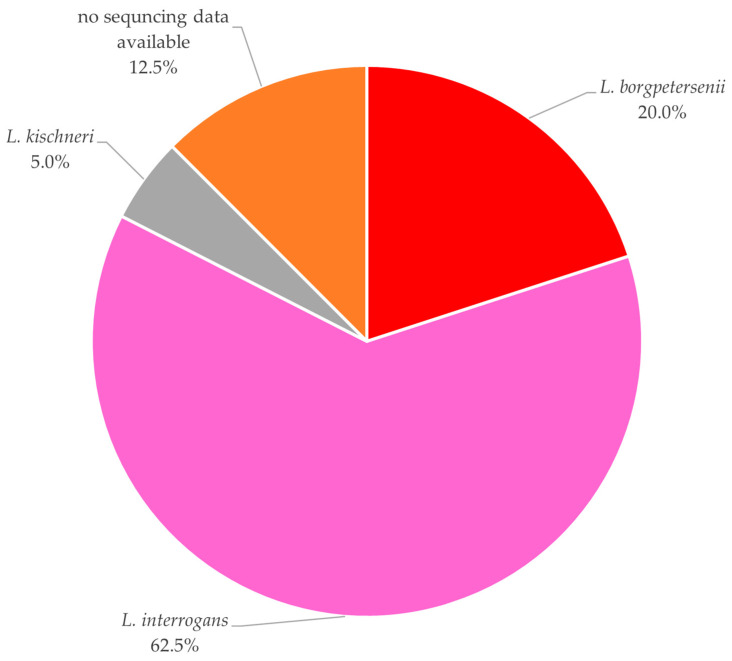
Distribution of *L*. species among samples testing positive for DNA of pathogenic *L*., based on BLASTn analysis. Percentages refer to the total number of pathogenic *L*. positive samples, including samples that could not be further characterized due to insufficient PCR products or sequencing data.

Among the 40 positive pathogenic *Leptospira* DNA samples, BLASTn analysis identified 25 (62.5%) as *L. interrogans*, 8 (20.0%) as *L. borgpetersenii*, and 2 (5.0%) as *L. kirschneri*. The remaining 5 samples (12.5%) could not be further characterized, either due to insufficient PCR product or inadequate sequencing quality (Figure 8).

For the samples positive for intermediate *Leptospira* DNA, all 7 sequences that yielded sufficient data for BLASTn analysis were identified as *L. fainei*.

**Figure 9:**
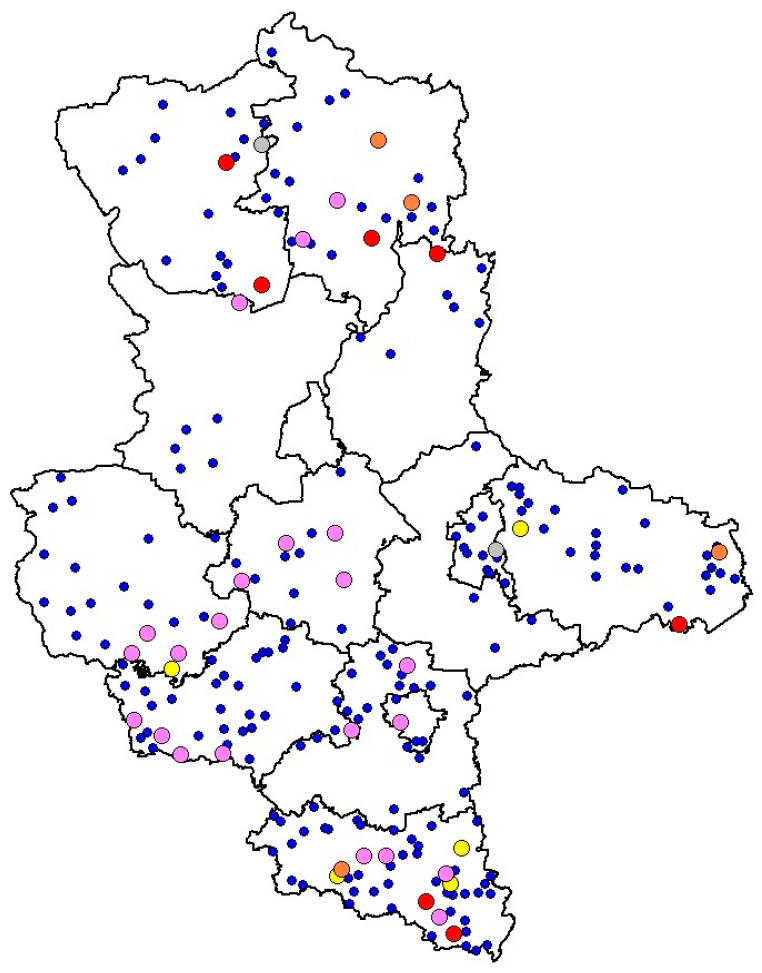
Spatial distribution of *L*. genotypes identified in wild boar kidney samples across the investigated administrative districts. Blue dots indicate PCR negative samples; colored dots indicate samples showing the highest nucleotide similarity to *L. interrogans* (pink), *L. borgpetersenii* (red), *L. kirschneri* (gray), and *L. fainei* (yellow), respectively. Orange dots represent PCR-positive samples (pathogenic or intermediate *Leptospira*) that did not yield sequence data of sufficient quality for BLASTn analysis.

The spatial distribution of the different *Leptospira* genotypes across the investigated districts is illustrated in Figure 9. The map provides an overview of the geographical occurrence of pathogenic and intermediate *Leptospira* DNA detected in wild boar kidney samples and highlights the distribution of identical genotypes among the different districts.

## 4. Discussion

Wild boar (*Sus scrofa*) are increasingly recognized as important reservoirs of zoonotic pathogens, owing to their wide distribution, high population densities, and close interactions with both domestic animals and humans [28]. In this context, the present study provides comprehensive molecular evidence of the circulation of pathogenic and intermediate *Leptospira* spp. in wild boar from Saxony-Anhalt, highlighting spatial, host-related, and seasonal patterns that deepen our understanding of *Leptospira* ecology in a key wildlife species.

In contrast to serological investigations, which have been extensively conducted in wild boar populations [17,20,29,30,31,32,33,34,35,36,37,38,39,40,41], molecular studies targeting pathogenic *Leptospira* DNA in kidney tissue remain scarce in Europe and particularly in Germany. Available studies from other European countries have reported prevalences ranging between approximately 10% and 13%, depending on the geographical context, sampling design, and diagnostic approach applied [17,30,31]. Comparable variability has also been described outside Europe, with prevalences of 3.4% and 3.2% reported in two independent investigations conducted in the United States of America [42,43], whereas a markedly higher prevalence of 10.3% was observed in wild boar in Japan [44].

In the present study, *Leptospira* DNA was detected in 3.7% of wild boar kidney samples, a prevalence that is notably lower than that previously reported by serological investigations conducted on the same population, where an antibody prevalence of 12.4% was observed [20]. This apparent discrepancy between molecular and serological findings is not unexpected. It has been described by numerous authors in both wild boar and pig studies [17,30,31,42,43,44,45,46,47] and reflects fundamental differences between the two diagnostic approaches. While serology provides evidence of previous exposure and immunological contact with *Leptospira* spp., PCR-based detection is indicative of current renal colonization and, consequently, of the potential for active shedding of the pathogen [48,49]. Furthermore, renal carriage of *Leptospira* spp. may be intermittent or present at low bacterial loads, influenced by host immunity, infection stage, and bacterial load, which can limit PCR sensitivity, particularly in chronically infected animals [50]. In contrast, antibodies may persist for months or even years after infection, leading to higher apparent prevalence in serological surveys [49]. Together, these factors may contribute to lower PCR-based prevalence compared to serological data and underline the complementary nature of both approaches when assessing the epidemiological role of wild boar as reservoirs of *Leptospira* spp.

The widespread detection of pathogenic *Leptospira* DNA across almost all investigated administrative districts suggests an endemic circulation of leptospires within the wild boar population of Saxony-Anhalt. The absence of positive samples in Magdeburg city is likely attributable to the lack of available samples from this district, while the absence of positives in Anhalt-Bitterfeld may similarly reflect the limited number of samples analyzed, with only ten kidneys examined.

A significatively higher prevalence of pathogenic *Leptospira* DNA was observed in the district of Salzlandkreis, indicating potential local environmental or ecological factors favoring transmission. Such spatial heterogeneity has been reported in other studies and may be associated with differences in landscape structure, agricultural practices, water availability, or density of wildlife hosts and livestock [51]. Additionally, differences in hunting practices and number of samples between districts could also contribute to the observed variation. Further detailed investigation of these factors is needed to better understand the drivers behind the regional differences in the prevalence of *Leptospira* species as well as antibodies against them. In contrast, intermediate *Leptospira* DNA was detected sporadically and without statistically significant spatial clustering, suggesting a more restricted or irregular circulation pattern.

No statistically significant differences in prevalence were observed between male and female wild boar for either pathogenic or intermediate *Leptospira* DNA, which is in line with several studies indicating that sex alone is not a major determinant of infection risk in wild boar [17,30,31,52]. In contrast, age was identified as a significant factor for pathogenic *Leptospira* infections. Sub-adult animals exhibited the highest prevalence, and statistical analysis confirmed a significant association between age class and infection status. This pattern has been reported by other authors and is often attributed to increased exposure during dispersal and social interactions, combined with an immature or still-developing immune response [42]. Young animals may have had insufficient exposure time, whereas adults could benefit from acquired immunity or selective survival. For intermediate *Leptospira*, no significant association with age was observed, despite a higher number of positive samples in sub-adults. This finding supports the hypothesis that intermediate species may play a less prominent role in persistent renal colonization or long-term maintenance within wild boar populations.

Seasonality emerged as an external factor influencing the prevalence of pathogenic *Leptospira* DNA. The significantly higher detection rate observed in autumn is consistent with previous reports describing seasonal peaks associated with increased rainfall, higher environmental humidity, and moderate temperatures that favor leptospiral survival in soil and water [48,49,53,54,55,56,57,58,59,60]. Additionally, as described by Calosi et al. [61], autumn coincides with increased wild boar activity, aggregation, and hunting pressure, potentially enhancing detection probability. No significant seasonal pattern was observed for intermediate *Leptospira*, further supporting the notion that these species may be less influenced by environmental conditions or may circulate at lower levels within the host population. Another reason could be that wild boar are less prominent reservoir hosts for intermediate *Leptospira* species.

However, further studies are needed to enable a more in-depth analysis of environmental factors (e.g., climate parameters, landscape characteristics, habitat types) and infection risk. The data in the present study refer only to the location where each animal was hunted, which does not necessarily correspond to where it lived or where infection was acquired. As demonstrated in recent studies [62], wild boar have increasingly adapted to anthropogenic environments, and intra-urban home ranges may vary widely, ranging from 1.3 to 64.6 km^2^. Such extensive and variable movement patterns make it difficult to reliably associate infection status with specific environmental or climatic parameters based solely on hunting locations.

Molecular characterization revealed *L. interrogans* (62.5%) as the predominant pathogenic species, followed by *L. borgpetersenii* (20.0%) and *L. kirschneri* (5.0%). As expected, these were similar patterns observed in a serological study conducted in the same wild boar population [20], confirming prevalence rates in this and other European studies, further suggesting *L. interrogans* as the most represented genotype [17,30,31].

While data on pathogenic *Leptospira* DNA in wild boar are limited, information on intermediate *Leptospira* is even scarcer. To the best of the authors’ knowledge, detection of intermediate *Leptospira* in wild boar kidneys have only been reported in Italy, specifically in the Liguria region and Tuscany, with prevalence rates of 0.49% and 3.14%, respectively. So far, no data are available from Germany. All positive samples sequenced in the current study belonged to the *L. fainei* genotype. *L. fainei* was first isolated from pigs, and specific antibodies have been detected in human sera in Australia [63,64], with human infections associated with febrile status reported in France and in two patients in Denmark [65,66]. To date, no data are available on *L. fainei* infections in Germany, neither in humans nor in animals.

In contrast, infections caused by pathogenic *Leptospira* species are well documented both in Germany and worldwide. Leptospirosis is the most globally widespread bacterial zoonosis, resulting in over 1 million human infections annually and nearly 60,000 deaths [49,67]. Traditionally, certain occupational groups such as farmers, veterinarians, hunters, slaughterhouse workers, or sewage workers have been considered particularly at risk. In recent decades, however, the risk profile has expanded. Increasingly, recreational and sports exposures are being identified as relevant sources of infection, particularly water sports in freshwater environments, such as canoeing and kayaking, rafting, cave tours, and triathlons [68,69,70,71].

Nevertheless, leptospirosis remains probably a largely underestimated disease, as it frequently presents with non-specific febrile symptoms that may go unrecognized or be misdiagnosed. Therefore, it is essential that diagnostic protocols also consider the possibility of infections with intermediate *Leptospira* strains, including *L. fainei*, particularly in regions where wildlife reservoirs may play a role in transmission. Considering wild boar behavior and their ability to inhabit anthropogenic environments, transmission between humans and wildlife is plausible, especially for people with relevant professional or leisure activities. As this is the first detection of intermediate *Leptospira* in German wildlife, further studies are needed to elucidate the epidemiology of this pathogen, which has the potential to cause severe infections in humans [64,65,66].

## 5. Conclusions

In conclusion, this study provides comprehensive molecular evidence confirming the role of wild boar as renal carriers of *Leptospira* spp. in Saxony-Anhalt, Germany. The detection of pathogenic *Leptospira* DNA in kidney samples highlights the contribution of wild boar to the maintenance and environmental dissemination of these bacteria, reinforcing their value as sentinels of *Leptospira* circulation within shared ecosystems. The widespread spatial distribution observed, together with the significant associations with sub-adult age class and autumn season, underscores the complex interplay between host biology, environmental conditions, and pathogen persistence.

Beyond pathogenic species, the molecular detection of intermediate *Leptospira* represents a particularly relevant finding. Notably, this study provides the first molecular evidence of *L. fainei* in wild boar kidneys in Germany, marking the first description of this intermediate species in the country at all.

From a One Health perspective, the presence of pathogenic and intermediate *Leptospira* spp. in apparently healthy wild boar has important implications for wildlife, livestock, and humans sharing the same environments, highlighting that infection and the possible shedding of the pathogen can occur without clinical signs. Overall, these findings underline the potential zoonotic risk of direct or indirect contact with wild boar, particularly the handling and preparation of potentially infected meat or organs and indicate the necessity of proper hygiene practices.

## Figures and Tables

**Table 1:** Oligonucleotide sequences of primers and probes used in this study.

Target Region	Primers/Probe	Sequence 5′-3′	Fragment Size	Reference
lipL32 gene for pathogenic *Leptospira* spp.	lipL32_Forward	AAGCATTACCGCTTGTGGTG	241 bp	[24]
	lipL32_Reverse	GAACTCCCATTTCAGCGAT		
	Probe sequence	AAAGCCAGGACAAGCGCCG (5′-FAM–labeled)		
gene for intermediate *Leptospira* spp.	Interm_Forward	GAGTAACACGTGGGTAATCTTCCT	153 bp	[25]
	Interm_Reverse	TTTACCCCACCAACTAGCTAATC		
	Probe sequence	CTGGGATAACTTT (5′-6-FAM–labeled)		
	Probe sequence	TCGGGTAAAGATT (5′-VIC–labeled)		
*rrs2* gene (cPCR amplification of pathogenic *Leptospira* spp.)	*rrs2*_Forward	CATGCAAGTCAAGCGGAGTA	541 bp	[26]
	*rrs2*_Reverse	AGTTGAGCCCGCAAGTTTTC		
*16S rRNA* gene (cPCR amplification of intermediate *Leptospira* spp.)	*16S rRNA*_Forward	AGTTGATCCTGGCTC	541 bp	modified by the authors
	*16S rRNA*_Reverse	GGCTGGATCACCTCCTT		

## Data Availability

The original data presented in this study are available on request.

## Supplementary Materials

The following supporting information can be downloaded at: https://www.mdpi.com/article/10.3390/ani16071025/s1, Table S1: Total number of wild boar kidneys examined in Saxony-Anhalt, hunted in 14 districts and tested for the presence of pathogenic *Leptospira* DNA. Table S2: Total number of wild boar kidneys examined in Saxony-Anhalt, hunted in 14 districts and tested for the presence of intermediate *Leptospira* DNA. ABI: Anhalt-Bitterfeld, BK: Börde, BLK: Burgenlandkreis, DE: Dessau-Roßlau, HAL: Halle (Saale), HZ: Harz, JL: Jerichower Land, MD: Magdeburg-city, MSH: Mansfeld-Südharz, SK: Saalekreis, SLK: Salzlandkreis, SAW: Salzwedel, SDL: Stendal, WB: Wittenberg, ST: Saxony-Anhalt. Table S3: Wild boar kidneys tested positive for pathogenic or intermediate *Leptospira* DNA with Ct values from the performed real-time PCR and geographical coordinates of the location where each animal was hunted. Table S4: Wild boar kidneys tested negative for *Leptospira* DNA with geographical coordinates of the location where each animal was hunted.

## Abbreviations

The following abbreviations are used in this manuscript:

PCR	Polymerase Chain Reaction
*L*.	*Leptospira*
DNA	Deoxyribonucleic Acid
CI	Confidence interval
df	Degrees of freedom
OR	Odds ratio
*p*	*p*-value
rRNA	ribosomal Ribonucleic Acid
cPCR	conventional Polymerase Chain Reaction
